# Newborn Screening Saves Lives but Cannot Replace the Need for Clinical Vigilance

**DOI:** 10.1155/2018/7217326

**Published:** 2018-07-02

**Authors:** F. Neemuchwala, M. Taki, E. Secord, S. Z. Nasr

**Affiliations:** ^1^Department of Pediatric Pulmonary Medicine, Michigan Medicine, Ann Arbor, MI, USA; ^2^Department of Allergy, Asthma and Immunology, Children's Hospital of Michigan Specialty Center, Detroit, MI, USA

## Abstract

Newborn screening for cystic fibrosis (CF) enables early diagnosis and treatment leading to improved health outcomes for patients with CF. Although the sensitivity of newborn screening is high, false-negative results can still occur which can be misleading if clinicians are not aware of the clinical presentation of CF. We present a case of a young male with negative newborn screen diagnosed for CF. He was diagnosed at 3 years of age despite having symptoms indicative of CF since infancy. The delayed diagnosis resulted in diffuse lung damage and poor growth.

## 1. Introduction

Cystic fibrosis (CF) is the most common autosomal recessive disease in Caucasians in the US, occurring in approximately 1 in 3500 newborns [1]. Newborn screening (NBS) for CF started as a pilot study in Colorado in 1980 [2] after the identification of elevated immunoreactive trypsinogen (IRT) in the dried blood spot of newborns with CF [3]. In 1991, shortly after the identification of the CF gene, an IRT/DNA technique was developed and implemented in Wisconsin, and by 2010, it was implemented nationwide [4]. States variably use either IRT/IRT, IRT/DNA, or IRT/IRT/DNA protocol, and although each protocol aims for optimal sensitivity with high specificity, false-positive and false-negative results still occur [5].

Newborn screening for CF is cost-effective and has led to improvement in health outcomes for patients with CF [6]. The Michigan Department of Health and Human Services (MDHHS) implemented IRT/DNA screening for CF NBS in Michigan in 2007. Newborns with an IRT level greater than 96 percentile for the day undergo additional DNA analysis using a panel of forty of the common CFTR (cystic fibrosis transmembrane conductance regulator) mutations. Newborns who test positive for 1 or 2 CFTR mutations are referred for sweat chloride testing which is considered the gold standard for the CF diagnosis [7]. The Hologic InPlex® CF 40 mutation panel (Wisconsin, USA) [8] was replaced by Luminex® xTAG CFTR 60 kit v2 (http://www.luminexcorp.com/cf, Luminex 100™ and Luminex 200™, Chicago, USA) in March 2016. The Luminex xTAG CFTR 60 kit v2 screens for 60 CFTR gene mutations and has a sensitivity between 54.5 and 95.9% depending on patient ethnicity [9].

We present a case of a 32-month-old boy with chronic cough, recurrent infections, and failure to thrive (FTT) with negative CF newborn screen and significant delay in the diagnosis of CF leading to diffuse bronchiectasis and FTT.

## 2. Case Report

A 32-month-old Middle Eastern boy was born full term at a community hospital in Michigan with birth weight of 3135 g (15.0 percentile). He had normal prenatal ultrasounds. He passed meconium at birth and had no other complications including prolong neonatal jaundice or dehydration. His CF NBS showed serum IRT 139 ng/ml and was negative for the 40 gene mutations panel. At 1 month of age, he developed a wet cough without any other symptoms. He was followed by his primary care provider (PCP), and no treatment was given at the time. His symptoms continued on and off until 1 year of age. At 1 year, the mother noticed increased frequency of productive cough, lack of appetite, and poor weight gain. His weight-for-age percentile ranged from 0.3 to 5.0. His stools were reportedly normal. He had no excessive sweating. He was referred to an outside asthma/allergy specialist for evaluation of asthma. He was prescribed budesonide without any improvement. He had frequent pharyngitis and otitis media that were treated with oral antibiotics that reportedly helped treat acute infection, but the cough persisted. He was also prescribed a H2 blocker for possible gastroesophageal reflux disease, but no improvement in symptoms was noted. Family history was negative for CF.

At 30 months of age, he was seen by his PCP for one week of cough and fever. He was treated with amoxicillin. His symptoms continued to worsen despite oral antibiotics, and he had two episodes of small-volume hemoptysis. He was subsequently admitted for community-acquired pneumonia and influenza B. Chest X-ray showed diffuse ill-defined opacities in the perihilar area and diffuse bronchiectasis. During the hospitalization, pediatric pulmonary consult was obtained. Given the negative NBS, it was stated that CF was unlikely and no sweat chloride test was recommended. He had a normal videofluoroscopic swallow study. Immunodeficiency workup revealed elevated immunoglobulin levels, protective vaccine titers, and normal lymphocyte counts and response to phytohaemagglutinin, concanavalin A, and pokeweed mitogen. HIV test was negative. Pediatric gastroenterology was consulted for failure to thrive and recommended to continue high-calorie diet. He was discharged home on augmentin.

Ten days following discharge, he was seen at the immunology clinic. He was noted to have digital clubbing, worsening tachypnea, and crackles. With the concerning physical exam findings, a sweat chloride test was done with a result of 90 mmol/L (normal 0–29 mmol/L; intermediate 30–59 mmol/L; abnormal ≥60 mmol/L) [7]. He was referred to pediatric pulmonary clinic the same day. He was then admitted and treated for a CF exacerbation. Throat culture grew *Pseudomonas aeruginosa* and methicillin-sensitive *Staphylococcus aureus* (MSSA). Fecal elastase-1 was <50 mcg E/g stool (normal >200 mcg E/g stool). Lab results including comprehensive metabolic panel and vitamin A and E levels were normal. He completed two weeks of cefepime and tobramycin.

**Figure 1:**
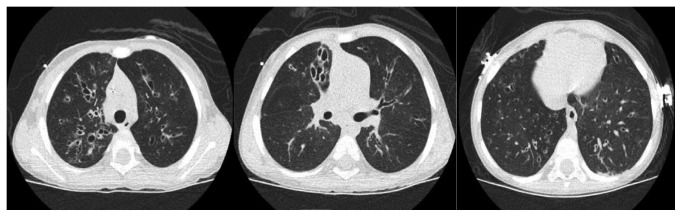
High-resolution computed tomography of the chest showing diffuse bilateral cylindrical/cystic bronchiectasis more pronounced in the upper lobes/right middle lobe than lower lobes with associated scattered areas of air trapping.

**Figure 2:**
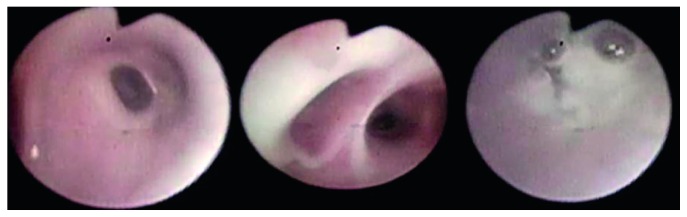
Thick purulent secretions on flexible bronchoscopy.

After notifying MDHHS with the false-negative NBS results, the blood spot that was available at the NBS lab was retested using the new and expanded mutation panel (60 mutations). He was found to be homozygous for R1066C (c.3196C > T; p.Arg1066Cys) mutation. His care was transferred to our CF center, as per parents' request. Two weeks later, he was admitted for worsening respiratory symptoms and treated for a CF exacerbation. Vitamin D level was low at 25 ng/ml (normal ≥30 ng/ml). High-resolution computed tomography of the chest showed diffuse bilateral bronchiectasis (Figure 1). Flexible bronchoscopy showed airway erythema and significant thick green secretions (Figure 2) that was positive for MSSA.

## 3. Discussion

This case highlights the need for considering the diagnosis of CF, despite normal NBS. When patients develop signs and symptoms that indicate CF, sweat chloride testing should be done despite normal NBS. NBS in CF leads to early diagnosis and improves health outcomes [10]. In Michigan, the IRT/DNA screening protocol screens for the common CFTR mutations in newborns following high IRT (sensitivity 96%) [11]. The Hologic InPlex CF molecular test that was used prior to March 2016 included 40 mutations and 4 variant polymorphisms in the CFTR panel. In March 2016, a recall of the Hologic system was issued nationwide due to 9 cases of false positives and 2 cases of technical issues [12]. Following the recall, the MDHHS implemented a new panel that included 60 gene mutations (Luminex xTAG CFTR 60 kit v2) [8].

In our patient, the IRT/DNA screening protocol was applied, which showed elevated IRT level (99.6 percentile). DNA analysis using the Hologic InPlex CF 40 mutation panel did not identify any mutation. After the positive sweat chloride test, the dried blood spot was retested for 60 gene mutation panel and was found to be homozygous for R1066C (c.3196C > T, p.Arg1066Cys) mutation. Based on the CFTR-2 database, this variant combination causes CF and pancreatic insufficiency, and there are 8 patients reported in the database with this combination [13].

There are many factors that account for a missed CF diagnosis after NBS [14]. These factors can range from a system's error to miscommunication of the results. Sometimes, a newborn screening specimen may not be obtained or the specimen may be inadequate or labelled incorrectly which are technical errors that can be avoided. In 2008, the range of unacceptable specimens reported by states to the National Newborn Screening Information System ranged from 0.06% to 10.02% [14]. On the other hand, if an adequate specimen is collected, having an IRT level below the cut-off value can result in false-negative screening. On occasions, uncommon mutations are present which may not be identified on the screening panel, in which case strong clinical vigilance should be maintained [14]. A few cases of delayed diagnosis after false-negative NBS for CF have also been reported previously [15–18]. In our patient, the NBS result was negative as per the Michigan NBS algorithm. However, the diagnosis was missed due to lack of thorough evaluation of clinical signs and symptoms, not having CF in the differential diagnosis from infancy and misleading interpretation of negative NBS test by the specialist.

Sweat chloride testing is considered the gold standard for diagnosis of CF [19]. Abnormalities in the sweat chloride concentration are due to the lack of functional CFTR in the sweat glands preventing reabsorption of sodium and chloride. CF NBS does not provide definitive diagnosis of the disease, and those with clinical symptoms suggestive of CF must undergo sweat chloride testing to confirm the diagnosis [20, 21].

Clinical presentation of CF can vary widely [15–18]. They can present with chronic respiratory symptoms such as chronic productive cough, bronchitis, and recurrent pneumonia leading to bronchiectasis. This occurs due to lack of functional CFTR protein leading to dehydration of mucus layer in the airways, increasing the viscosity, impairing mucus clearance, and causing recurrent infections [21]. Patients can also present with chronic gastrointestinal manifestations including acute or recurrent pancreatitis, pancreatic insufficiency causing steatorrhea and failure to thrive, neonatal cholestasis, and meconium ileus [21]. Poor growth can in turn negatively impact lung function in these patients and lead to progressive decline over time [22]. Hence, NBS for CF offers the advantage of early diagnosis and has added benefits of improved growth, lung function, and survival [10]. However, this is a screening program and false-negative cases can result with the test.

In summary, this case highlights some of the limitations of NBS in CF. NBS is designed to identify patients with the disorder before they present clinically. It also gives the opportunity to screen siblings and family members and provide genetic counselling. As a screening program, there will be false-negative cases which lead to delayed diagnosis. In our patient, delayed diagnosis led to diffuse lung damage, bronchiectasis, poor growth, and FTT. It has also impacted the family's emotional status due to the stress of dealing with a diagnosis that has long-term consequences. Maintaining a high index of clinical suspicion when presented with characteristic clinical features is crucial for early diagnosis and treatment in CF.
